# An Extended Neck Position is Likely to Produce Cervical Spine Injuries Through Buckling in Accidental Head-First Impacts During Rugby Tackling

**DOI:** 10.1007/s10439-024-03576-z

**Published:** 2024-07-14

**Authors:** Pavlos Silvestros, Ryan D. Quarrington, Ezio Preatoni, Harinderjit S. Gill, Claire F. Jones, Dario Cazzola

**Affiliations:** 1 Department for Health, University of Bath, Claverton Down, Bath, BA2 7AY UK; 2 Centre for Analysis of Motion and Entertainment Research and Application, University of Bath, Bath, UK; 3 Centre for Health and Injury and Illness Prevention in Sport (CHi2PS), University of Bath, Bath, UK; 4 Department of Mechanical Engineering, University of Bath, Bath, UK; 5 Centre for Therapeutic Innovation, University of Bath, Bath, UK; 6 Centre for Orthopaedic & Trauma Research, Faculty of Health and Medical Sciences, The University of Adelaide, Adelaide, Australia; 7 School of Electrical and Mechanical Engineering, The University of Adelaide, Adelaide, Australia

**Keywords:** Contact sport, Rugby, Spine, Injury prevention, Injury mechanisms, Musculoskeletal modeling

## Abstract

Catastrophic cervical spine injuries in rugby often occur during tackling. The underlying mechanisms leading to these injuries remain unclear, with neck hyperflexion and buckling both proposed as the causative factor in the injury prevention literature. The aim of this study was to evaluate the effect of pre-impact head–neck posture on intervertebral neck loads and motions during a head-first rugby tackle. Using a validated, subject-specific musculoskeletal model of a rugby player, and computer simulations driven by in vivo and in vitro data, we examined the dynamic response of the cervical spine under such impact conditions. The simulations demonstrated that the initial head–neck sagittal-plane posture affected intervertebral loads and kinematics, with an extended neck resulting in buckling and supraphysiologic intervertebral shear and flexion loads and motions, typical of bilateral facet dislocation injuries. In contrast, an initially flexed neck increased axial compression forces and flexion angles without exceeding intervertebral physiological limits. These findings provide objective evidence that can inform injury prevention strategies or rugby law changes to improve the safety of the game of rugby.

## Introduction

The rugby tackle is associated with a high proportion of head, neck, and shoulder injuries [3, 14, 52], generating the highest overall percentage of injuries in the sport [55]. Over the last decade, epidemiological and biomechanical injury research in rugby has primarily focused to reduce the risk of concussion [50–52]. Rugby tackling events are also responsible for more than 30% of all severe cervical spine injuries in the sport [4]. Although severe neck injuries are rare (0.04/1000 player-match-hours) [18] compared to tackle-related concussion (20.9/1000 player-match-hours) [54], the consequences of the former are typically substantial and life altering [37]. There is currently a lack of understanding regarding the mechanisms of neck injuries associated with the most common rugby tackling techniques, and it is possible that policy changes seeking to mitigate the risk of mTBI may inadvertently increase the risk of cervical spine injury. Therefore, there is a pressing need to identify the factors that lead to severe rugby neck injuries so that changes to rugby tackling rules are appropriately informed. Tackler neck injuries occur more frequently during “front-on” and “active shoulder” tackles [13, 52] in which the tackler is more likely to make contact with the ball carrier’s body, and assume a more crouched position. This configuration increases the likelihood of misdirected “head-first” tackles, where contact occurs between the tackler’s head and the ball carrier’s torso, leading to head and neck injury [49]. Such information is key to identifying the tackle characteristics associated with higher injury risks, especially when new tackle laws are proposed, but the key mechanisms underlying tackle-related neck injuries have not yet been described.

Bilateral facet dislocations (BFDs) in the lower cervical spine (i.e., between the fourth and seventh cervical vertebrae [C4 to C7]) are the most commonly observed catastrophic neck injuries in rugby [25]. Two competing theories regarding the mechanisms underlying BFD are described in the clinical and injury biomechanics literature [32]: “buckling”; and, “hyperflexion.” Buckling is characterized by concurrent upper neck extension and lower neck flexion, and has been observed during in vitro experiments in which an axial compression load is applied to the neck [2, 19, 20, 33, 57]. In these experiments, BFD occurred at the spinal level of transition from flexion to extension following buckling, and complex combinations of bending, compression, and shear loads were observed at the level of injury. Head–neck hyper-flexion is described as a rapid and/or forceful posterior-to-anterior head rotation that causes supraphysiologic intervertebral flexion, failure of the posterior ligaments, and BFD [17]. One experimental study was able to produce BFD in functional spinal units via inertially induced hyperflexion (i.e., hyperflexion caused by the forward momentum of the head after the trunk momentum is arrested) [22], but lower cervical spine BFD has not occurred in whole head–neck specimens through hyperflexion alone (i.e., hyperflexion caused by a flexion inducing load placed on the head or neck) [19, 20, 31, 33].

Despite consensus among the injury biomechanics community that catastrophic cervical spine injuries are unlikely to occur due to head-neck hyperflexion [32], it has been suggested that it may be premature to abandon the hyperflexion mechanism as a contributor to rugby-related neck injury [10]. “Buckling” has only been observed during in vitro experiments, where the spine is devoid of musculature that provides dynamic stability, global stiffness, and contributes to vertebral kinematics [31], and has not been supported by in vivo observation of real-world injuries. In fact, player recollections [1] and video analysis of rugby cervical spine injuries have supported hyperflexion being the primary loading mechanism. These studies are based on qualitative outcomes and may suffer from considerable subjectivity, so a realistic and accurate analysis of the spinal loads experienced during head-on misdirected tackles in rugby is required.

Computer models of head impact are able to recreate, with high fidelity, the internal (i.e., muscle and joint contact forces) and external loading conditions of the event, and estimate the resulting neck kinematics [7, 8, 11, 16, 35]. Nightingale et al. (2016) performed computer simulations of experimental data [33] and demonstrated decoupling between externally observed head and neck kinematics, and the internal dynamic response of the spine during axial loading injuries [20, 33, 57]; buckling was the primary injury mechanism. More recently, computer simulations using musculoskeletal models have strengthened the theory that muscle forces affect resulting head and neck dynamics during injurious scenarios (inertial and axial impacts) [11, 28, 35]. However, seemingly arbitrary levels of simulated muscle activation, and the resulting muscle forces, have been used in these studies, limiting their direct applicability to real-world scenarios.

A computational investigation of rugby-specific impact scenarios has the potential to provide new insights into the predominant cervical spine injury mechanism observed during tackling. Computational approaches, by replicating the high-risk impact scenarios associated with catastrophic neck injuries occurring in rugby gameplay situations, enable researchers to relate them back to applied aspects of the sport, including player tackling technique and the governing laws of the game.

The aim of this study was to conduct a computer simulation study to investigate the effect of pre-impact neck position on intervertebral loads and motions during misdirected “head-first” rugby tackles. We hypothesized that the simulated mechanical response would support buckling as the primary mechanism of catastrophic rugby-related neck injuries.

## Materials and Methods

### Experimental Data

#### In Vivo Data Collection

One academy-level front-row rugby player (male, 22 years, 1.82 m, 113.7 kg) participated in this study. Ethical approval was obtained from the Research Ethics Approval Committee for Health at the University of Bath (Approval Number: EP 15/16 131) and the participant provided written informed consent prior to data collection. The experimental data collection on the participant was performed in accordance with the Declaration of Helsinki. Kinematics and electromyography (EMG) signals at the instant of tackle impact were used to inform the initial conditions of the model during the computer simulations. Full-body kinematics (Oqus, Qualysis, Sweden) was collected at 250 Hz, and bilateral EMG recordings (Trigno, Delsys, USA) of the sternocleidomastoid and upper trapezius muscles were collected at 2500 Hz during laboratory-based staged tackling trials with a custom tackle simulator [44]. The EMG signals were band-pass filtered (10-250 Hz Butterworth filter; maintaining 97% of signal power), full wave rectified, low-pass filtered at 6 Hz [26] with the same filter, then amplitude normalized to the maximum recorded value identified in maximum voluntary contraction trials to create EMG linear envelopes. Custom functions in Matlab R2017a (The Mathworks Inc., Natick MA, USA) were used for kinematic and EMG data processing. For the musculoskeletal simulations explained below, only a single representative experimental trial was chosen to provide initial conditions and to drive the musculoskeletal simulations. This approach was taken to reduce any information losses of the signal by averaging experimental data.

High-resolution isotropic (1 mm3 voxel) T1-weighted magnetic resonance images (MRI) (Skyra, Siemens, Germany) of the participant head and upper torso were obtained on the same day as experimental data collection [46]. The MRI images were semi-automatically segmented and used to create a participant-specific musculoskeletal model [46] as described.

#### In Vitro Data Collection

A head and neck assembly of an anthropometric test device (ATD; Hybrid III 50th percentile male, Humanetics, Germany), rigidly mounted to a steel frame at 1.5 m high [48], and the tackle simulator (i.e., 40 kg punching bag) [44], were used to estimate the external load experienced by players during misdirected rugby tackle impacts to the head. The ATD was equipped with a six-axis load cell located at the head–neck interface, and used to measure (1000 Hz) the forces resulting from contact between the tackle simulator and the ATD head. The force–time profiles were used to inform the impact conditions applied during the computer simulations. Impacts were generated by the tackle simulator contacting the ATD assembly at two speeds (2.0–2.5 m/s and 3.1–3.6 m/s) [48], representing the momentum change experienced during live tackles [5].

#### Musculoskeletal Simulations

## Musculoskeletal Model

The population-specific male-forward Rugby Model [6] was used as the baseline model for this study. Modifications were carried out in OpenSim 4.0 [9] and consisted of the (i) inclusion of hyoid muscle group to improve physiological fidelity [29], (ii) personalization of cervical vertebral dimensions using MRI, and (iii) integration of wrapping surfaces to better replicate muscle-tendon units (MTU) lines of action in the cervical spine. The neck region (C1/C7) of the musculoskeletal model was scaled in each dimension (height, width, and depth) based on anatomical measurements of the participant’s cervical vertebrae from the segmented MRI images. MTU attachment sites were not changed with respect to the population specific model [6, 53], due to difficulties in identifying muscle attachment locations in the MRI. The remaining model segments were linearly scaled based on anatomical motion capture markers. The dynamics of each sub-axial cervical spine joint (C2/C3 to C6/C7) was modeled using validated six degrees-of-freedom (6 DOF) Kelvin–Voigt bushings [47]. These bushings characterized the response of the passive structures around the intervertebral cervical joints under high energy impacts. The participant-specific wrapping surfaces (based on MRI) added were (i) a cylinder anterior to the lower cervical spine registered to the C6 vertebra [24]; (ii) a sphere originating at, and registered to, the C2 vertebra; iii) two bilateral cylinders at the posterior of the upper cervical spine registered to the C2 vertebra; and iv) two bilateral tori at the posterior lower cervical spine registered to the C7 vertebra. All wrapping surfaces were constrained to move with their registered bodies as detailed in Silvestros et al. [46]. The newly developed model is available from the SimTK repository (https://simtk.org/projects/csibath).

### Neck Angle Conditions

To examine the effect of initial neck position on intervertebral loading during head-on tackle impacts, the model’s sub-axial neck angle (C2/T1) was altered, with intervertebral contribution dictated by validated kinematics constraints, in 5° increments in all planes of motion: the sagittal plane from 30° of extension to − 30° of flexion (13 conditions); frontal plane from 0° (neutral) to − 10° of lateral bending (3 conditions); and transverse plane from 5° to 15° of axial rotation (3 conditions). This resulted in 117 unique initial neck angle configurations. The angle ranges selected were informed by kinematic measurements of experimental tackling trials of university and professional level rugby players [23]. The angle of the upper cervical spine (C0/C1 to C1/C2) was not varied but always initialized to 18° extension, equivalent to the in vivo experiment. The upper cervical spina angle was chosen to replicate a more ”head-up” position of the tackler, which is widely coached for improved tackle technique (rugbysmart.co.nz) [15, 39]. The same pre-impact head and neck angular velocities were prescribed for each unique initial neck angle configuration and the values were taken from the in vivo experimental tackling trial.

### Muscle Activations

**Fig. 1:**
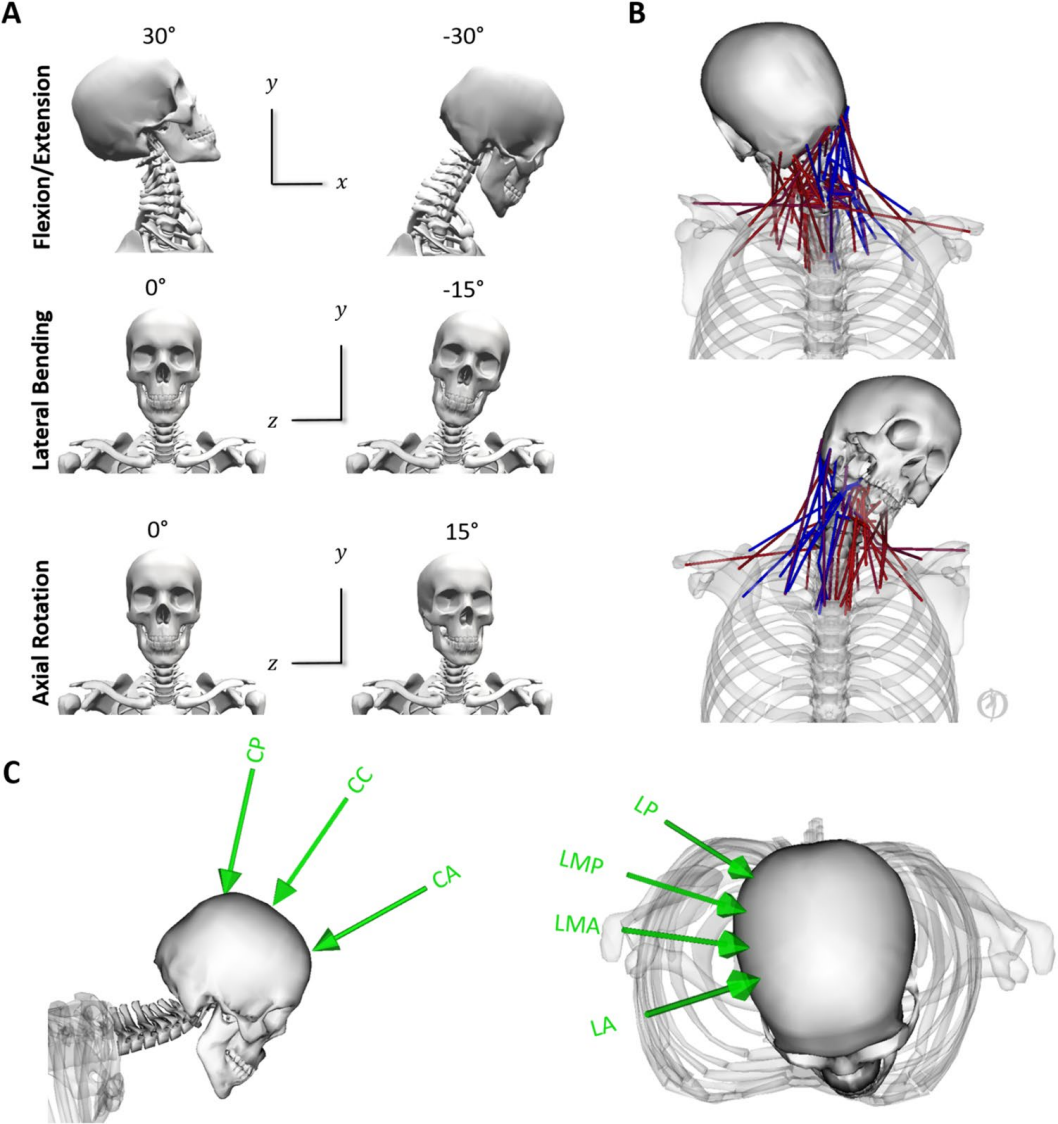
A–C—**A** Close-up view of the scaled MRI-informed OpenSim model’s head and neck region with reference views of maximal ranges of motion tested in the simulations (muscles and wrapping surfaces removed for clarity of the cervical spine structure). Anteroposterior shear and Lateral Bending are defined by the X axis. Compression and Axial Rotation are defined by the Y axis. Lateral Shear and Flexion/Extension are defined by the Z axis. **B** Neck muscle activation pattern estimated using EMG-assisted optimization from staged experimental tackling and used across all simulations. During the staged experimental trials, the tackle was taken on the right shoulder, which can be seen by the different levels of the model’s muscle activations (red—maximum, blue—minimum). **C** Cranial (left) and lateral (right) loading conditions applied to the skull (*CA *cranial anterior; *CC* cranial central; *CP* cranial posterior; *LP* lateral posterior; *LMP* lateral mid-posterior; *LMA* lateral mid-anterior; *LA* lateral anterior).

The same activation scheme (Fig. 1B) was used for all muscle groups for all simulations. This activation scheme was estimated using an EMG-assisted neuromusculoskeletal model [46] to minimize the error between experimental and simulated joint moments and muscle activations during the same in vivo experimental trial used to inform initial angular velocities. The estimate of the model’s 96 muscle activation patterns was solved using the Calibrated EMG-Informed Neuromusculoskeletal Modeling (CEINMS) OpenSim Toolbox [38] that minimized the cost function (Eq. 1):

1 $${\text{F}} = \alpha {\text{E}}_{{\text{M}}} + \beta {\text{E}}_{{{\text{e}}^{{2}} }}^{{\text{P}}} + \gamma {\text{E}}_{{\text{e}}},$$

where EM was the sum of the squared differences between the estimated and net joint moments from the inverse dynamics (sagittal and frontal plane moments of the C0–C1 through

to C6–C7 joints), ${\text{E}}\left( {{\text{P}}_{{{\text{e}}^{2} }} } \right)$ was the sum of the squared synthesized activations for all MTUs, and Ee was the sum of the differences between the adjusted model activations and experimental EMG recordings. Factors α, β, and γ were non-negative weightings for each term of the cost function. Activation dynamics were characterized by a critically damped linear second-order differential system [26, 38]. It was assumed that the MTU tendons of the model were stiff (i.e., not compliant) due to their short length and expected function in the neck. To provide a reasonable and physiologically plausible muscle recruitment pattern, muscle activation values were selected from the instant of tackle impact during the staged tackle trial then applied to the musculoskeletal model as preactivation and remained constant for the duration of the 50 ms forward dynamic simulations. Constant activations were selected to represent muscle preactivation as cervical spine reflex times exceed 50-60 ms [12, 30, 45], reducing the effect of active neck muscle modulation during short duration events.

### Loading Conditions

A “head-first” impact was defined as the incorrect tackle technique, where the tackler misaligns the position of their head, neck, and torso resulting in direct contact of their head with the oncoming attacking player. To replicate a range of possible head impact locations and loading vectors during accidental head-on tackles, seven loading conditions were defined for the simulations (Fig. 1C).

The load vector was defined from the point of application of the force to the C1 joint center (Fig. 1C: CA, CC, CP). The points of application and direction of contact forces were calculated in Matlab R2017a (MathWorks Inc., Natick, MA, USA) using the model skull geometry as reference. Three points of force application were defined on the cranial midline of the skull: vertex, posterior to vertex (near the lambda or ”crown”), and anterior to vertex. Four points of force application were defined on the right lateral skull, with an inferolateral direction representing more oblique impacts (Fig. 1C: LP; LMP; LMA; LA) toward the C1 joint center. All points of application and directional vectors were expressed with respect to the model’s skull coordinate reference system.

The magnitude and loading rate (80 kN/s) for each condition were acquired from the in vitro experimental trials [48]. The loading rate of these tests was an order of magnitude lower than the rate for which the musculoskeletal model bushing elements were validated (800 kN/s). However, it has been shown that the stiffness response of intervertebral discs does not change considerably above a rate of 75–90 N/s [30, 42] therefore the bushings used in the model were deemed valid for the loading conditions tested.

### Forward Dynamics Simulations

**Fig. 2:**
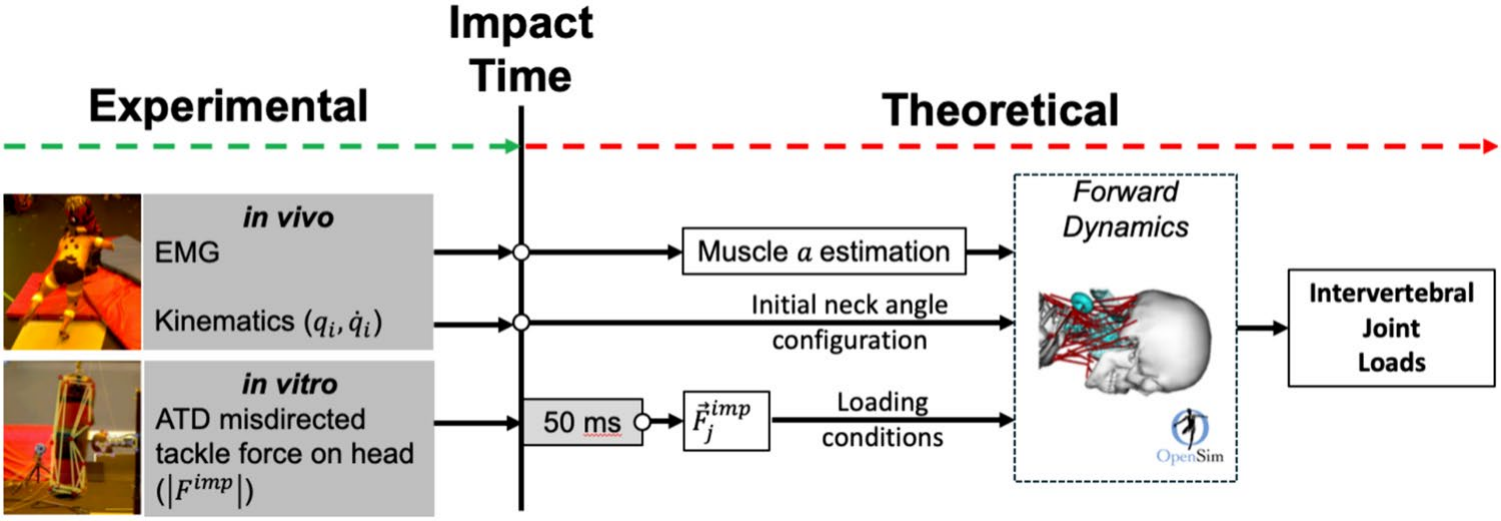
Workflow of integrated experimental and theoretical framework used to investigate cervical spine injury mechanism in rugby tackles. Experimental: in vivo data (neck muscle EMG and joint angles and velocities) were collected during stage tackling laboratory trials using a tackle simulator (mass = 40 kg, velocity = 3 m/s). In vitro data (force magnitude and loading rate) were collected from the Anthropometric Test Device (ATD) during simulated misdirected impacts to the head. Theoretical: for each of the 819 simulations, an initial neck angle configuration combining Flexion/Extension, Lateral Bending, and Axial Rotation angles (qi, *n* = 117) taken from ranges in the literature was prescribed to the model. In vivo data at the time of impact were used to inform then initial neck joint angular velocities (qdot) and joint angles of the torso and upper limbs. Level of neck muscle activations (a) at the time of impact derived from EMG-assisted analysis of the staged tackling trial were applied to the model’s muscles to be constant throughout the 50 ms simulations. For each initial neck angle configuration (qi), external loading conditions were applied (F^I^mp, *n* = 7) replicating different impact locations on the head at two different speeds. The points of application and direction of the loading conditions were defined using the model’s skull geometry in Matlab. The magnitude and loading rate characteristics were taken from the first 50 ms of the in vitro ATD impact forces.

A total of 819 simulations (117 neck angle configurations ∗ 7 loading conditions ∗ 1 loading rates) were performed using the OpenSim Forward Dynamics Tool (Max Step Number = 2000, Min Step Size = 1e^−8^, Max Step Size = 1, Error Tolerance = 1e^−6^, Output Precision = 6), each with a duration of 50 ms commencing at initial force application after the initialization of muscle forces. This duration was selected because in vitro head–neck impact experiments have detected neck injury within this time frame [33]. The simulations were not performed past the peak of the applied load as multibody models are unable to simulate tissue deformation, and thus are not expected to reliably predict the response in such conditions. The OpenSim model used in the simulations (see above) did not include any active kinematic constraints across the cervical joints, and the cervical spine dynamics was modeled via validated six degrees-of-freedom bushings [47]. All simulations that generated a C1-to-C6 flexion angle exceeding the chin-to-chest threshold established in in vitro studies (40°) were deemed invalid. The following parameters were obtained from each valid simulation: peak compressive force, peak anteroposterior shear force, and peak flexion bending moment, at each of the C3/C4 to C6/C7 joints (Fig. 2).

## Results

**Fig. 3:**
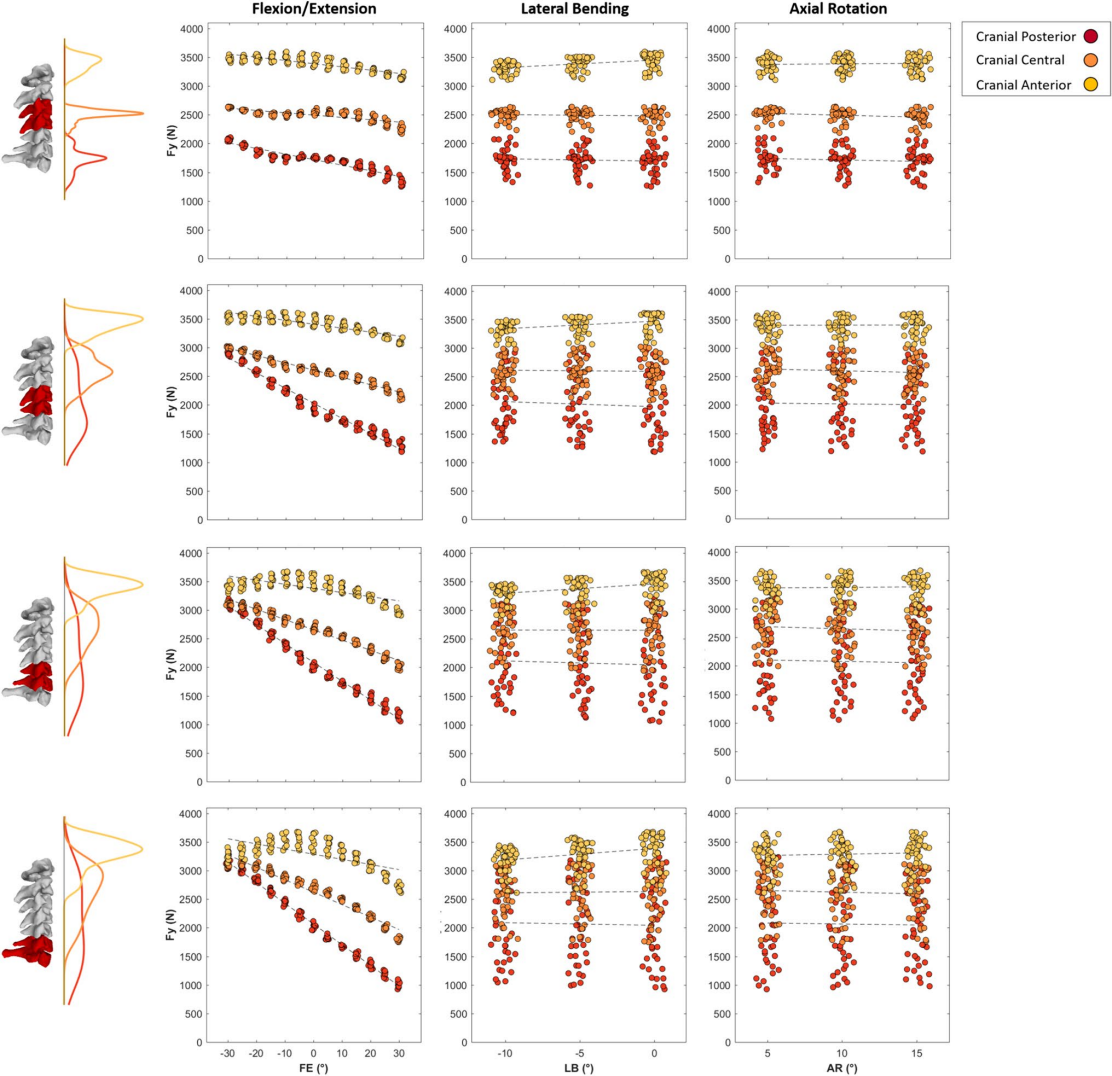
Maximal compressive joint loads (Newton) of C3–C4 (top row) to C6–C7 (bottom row) intervertebral joints plotted against 5^◦^ changes in Flexion(−)/Extension(+) (left column), Lateral Bending (center column), and Axial Rotation (right column) during the cranial loading conditions (Cranial Posterior, Cranial Central, and Cranial Anterior). Kernel density estimate plots to the left of the subplot rows represent the frequency distribution density of the maximal joint loads on the vertical axes for each loading condition. First-order polynomial lines of best fit are plotted to highlight the effect of joint angle on compressive joint loads for each loading condition (dashed lines). In each subplot, data points are spread slightly in each 5^◦^ bin on the horizontal axes for better visualization.

**Fig. 4:**
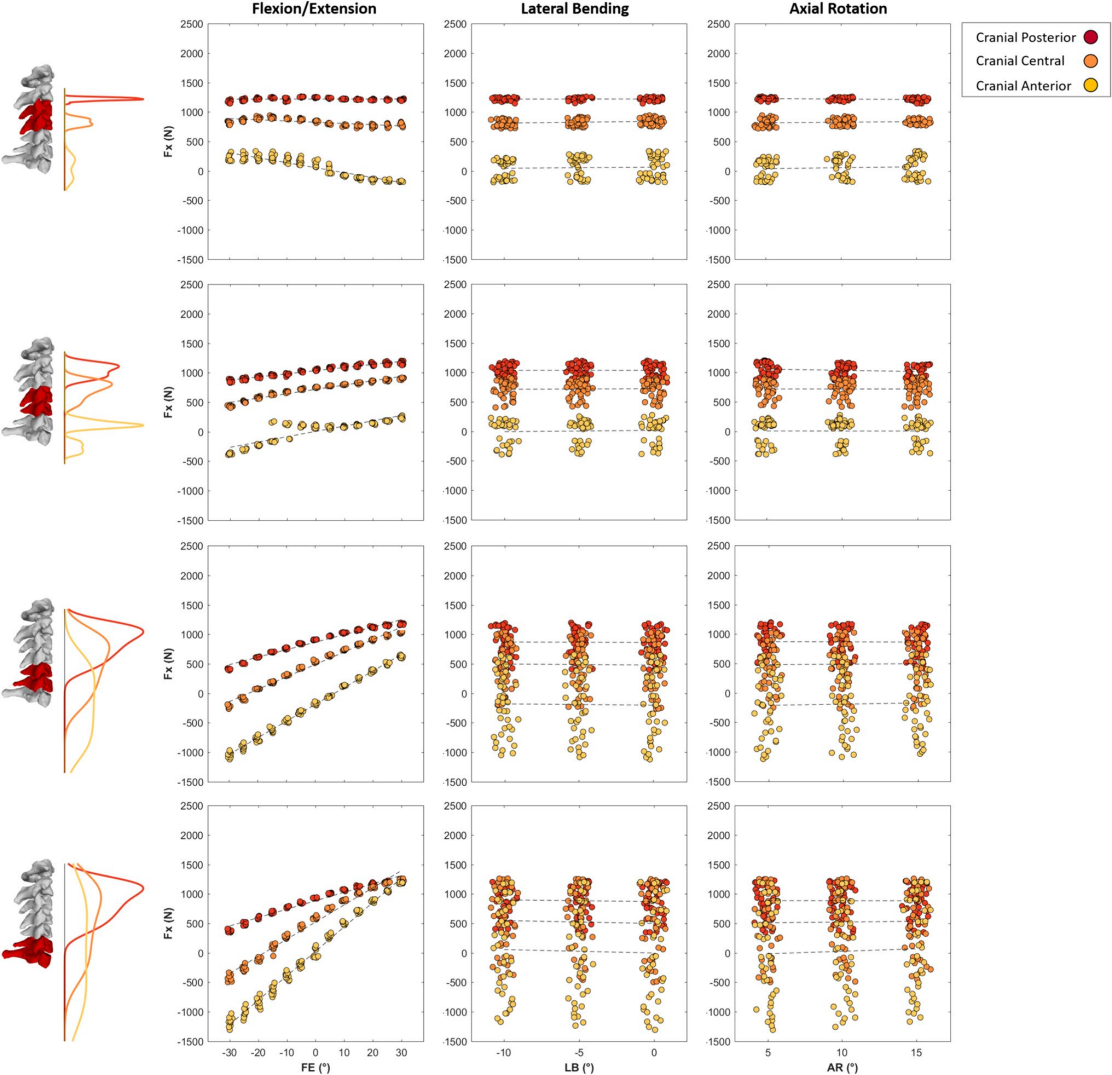
Maximal anteroposterior shear joint loads (Newton) of C3/C4 (top row) to C6/C7 (bottom row) intervertebral joints plotted against 5^◦^ changes in Flexion(−)/Extension(+) (left column), Lateral Bending (center column), and Axial Rotation (right column) during the cranial loading conditions (Cranial Posterior, Cranial Central, and Cranial Anterior). Kernel density estimate plots to the left of the subplot rows represent the frequency distribution density of the maximal joint loads on the vertical axes for each loading condition. First-order polynomial lines of best fit are plotted to highlight the effect of joint angle on compressive joint loads for each loading condition (dashed lines). In each subplot, data points are spread slightly in each 5^◦^ bin on the horizontal axes for better visualization.

**Fig. 5:**
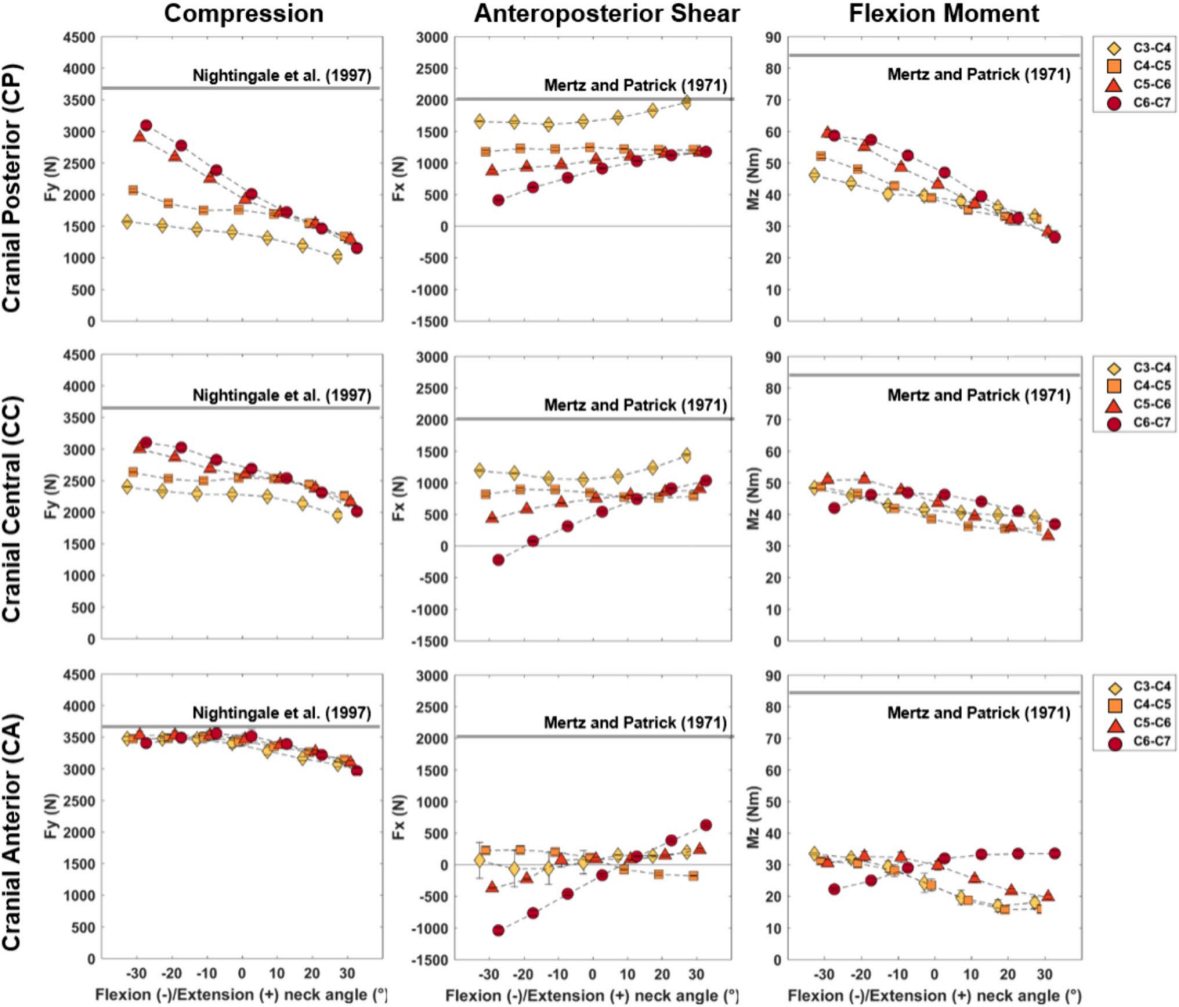
Mean and standard deviation values for maximal compression (left column), anteroposterior (center column), and flexion moment (right column) of all initial neck angle conditions plotted against changes in neck flexion (negative) and extension (positive) angles for cranial loading conditions (*CP* cranial posterior, *CC* Cranial Central, and CA cranial anterior). Estimated injury thresholds from the literature for the entire cervical spine are also presented with the horizontal lines for compression and anteroposterior shear. Flexion moment failure thresholds have been identified to be larger than 150 Nm for the intact cervical spine thus subjective thresholds of “Pain” and “maximum voluntary contraction” are presented.

**Fig. 6:**
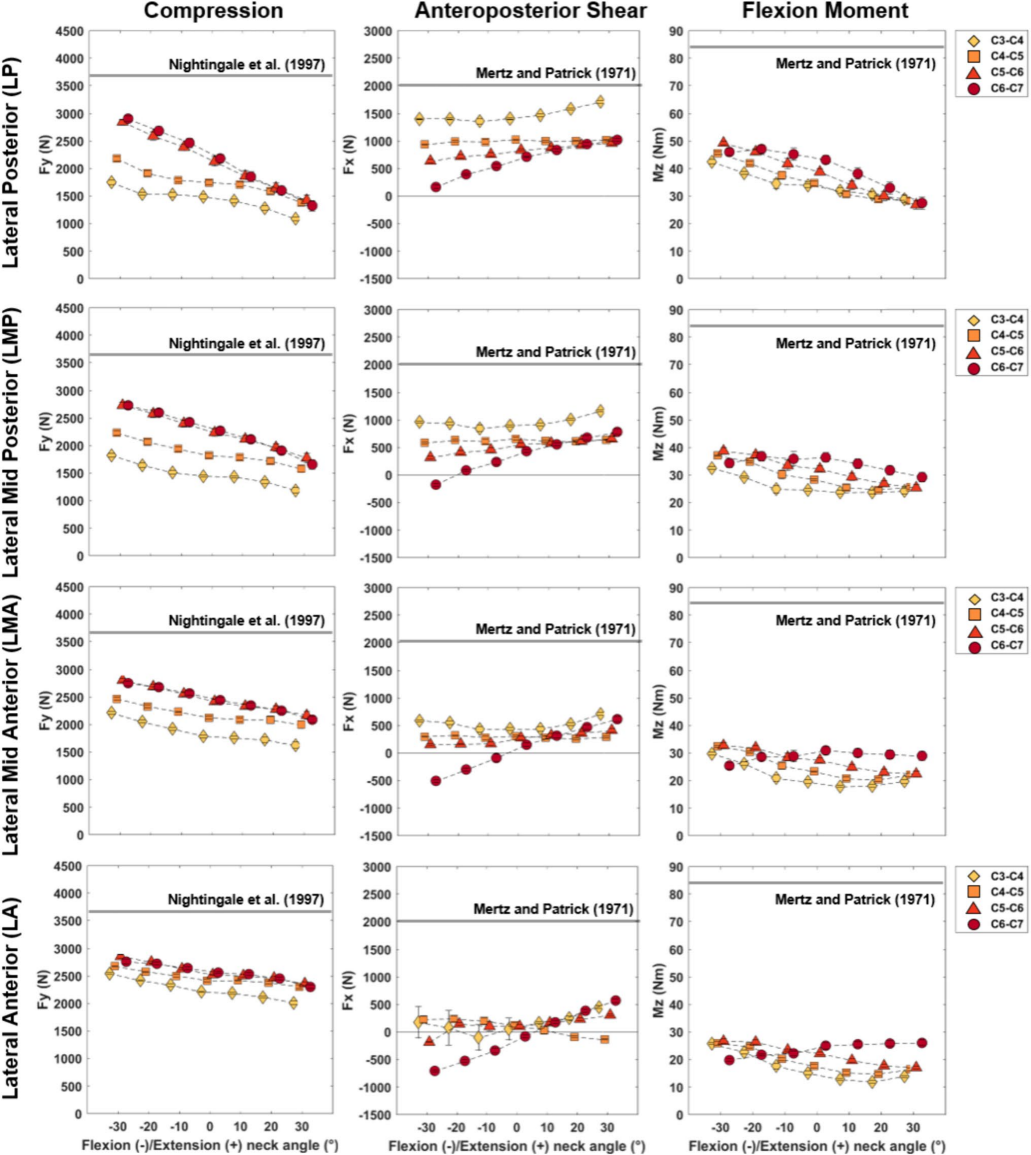
Mean and standard deviation values for maximal compression, anteroposterior, and flexion moment of all initial neck angle conditions plotted against changes in neck flexion (negative) and extension (positive) angles for lateral loading conditions (LP Lateral Posterior, *LMP* lateral mid-posterior, *LMA* lateral mid-anterior, and *LA* lateral anterior). Estimated injury thresholds from the literature for the entire cervical spine are also presented with the horizontal lines for compression and anteroposterior shear. Flexion moment failure thresholds have been identified to be larger than 150 Nm for the intact cervical spine thus subjective thresholds of “Pain” and “maximum voluntary contraction” are presented.

Joint loads were more sensitive to changes in the initial neck flexion angle than lateral bending or axial rotation, across all loading conditions and vertebral levels (Figs. 3 and 4). In general, peak compressive force was larger in the lower cervical spine (C5–C7) when the neck was flexed (Figs. 5 and 6), whereas peak anteroposterior shear force was higher in the upper cervical spine (C3/C4) and varied in magnitude across the spinal levels for the various neck angles and applied load directions.

The initial lateral bending and axial rotation pose of the neck did not substantially affect the magnitudes of peak compressive (Fig. 3) or anteroposterior (Fig. 4) joint force for each of the three ”cranial” impact locations. Compressive joint force increased as initial neck position transitioned from an extended (30°) to a flexed (− 30°) position (see black versus red curves in Fig. 8—Column 1), apart from lower spine levels in the cranial anterior loading direction (Fig. 3). The largest increase was seen in the cranial posterior loading direction during which lower cervical spine (C6/C7 and C5/C6) compressive loading increased by approximately 50% (from 2100 to 3200 N) in the − 30°flexed condition, compared to neutral (0°) (Fig. 3—Column 1 Rows 3 and 4, and Fig. 5—Column 1 Row 1). Lateral posterior impacts (LP and LMP) also resulted in increased compressive joint loading of up to 30% (from 2200 to 2900 N) (Fig. 6). In anterior loading conditions (CA, LMA, and LA), initial neck flexion had a smaller effect with compression force increasing less than 500 N (20%) from when the neck was extended (Figs. 5 and 6). Anteroposterior shear force changed direction from anterior to posterior for the lower vertebrae (Fig. 8—Column 2) as the initial neck flexion angle increased (Figs. 4 and 6—Column 2). This was more evident at the C5/C6 and C6/C7 joint levels, and during anterior loading of the skull (CA, LMA, and LA), where anterior shear loads of approximately 600 N during extension changed to posterior loads of 1000 N in flexion. Posterior loading conditions (CP, CC, LP, and LMP) resulted in anterior shear loading across the initial neck angles at all vertebral joint levels except C6/C7 in the most flexed conditions (Figs. 5 and 6—Column 2).

Peak flexion moments increased up to 60 Nm (Figs. 5 and 6—Column 3) for the upper cervical spine levels as the initial neck angle approached − 30°of flexion (see black versus red curve in Fig. 8—Column 3). This increase with neck flexion angle was more noticeable for the posterior loading conditions (CP and LP). Lower spinal levels showed an opposite trend in CA, LA, and LMA (Figs. 5 and 6). Peak flexion moments were larger in the lower than the upper cervical spine, for the majority of initial neck angles, when the spine was loaded at the posterior (CP and LP). Lateral shear displayed the lowest joint load magnitudes (¡ 1000 N). As left lateral bending angle increased left shear loads in the lower cervical spine (C5/C6 and C6/C7) also increased during cranial impacts. Lateral loading conditions (which were on the right side of the skull) increased the left lateral loading.

**Fig. 7:**
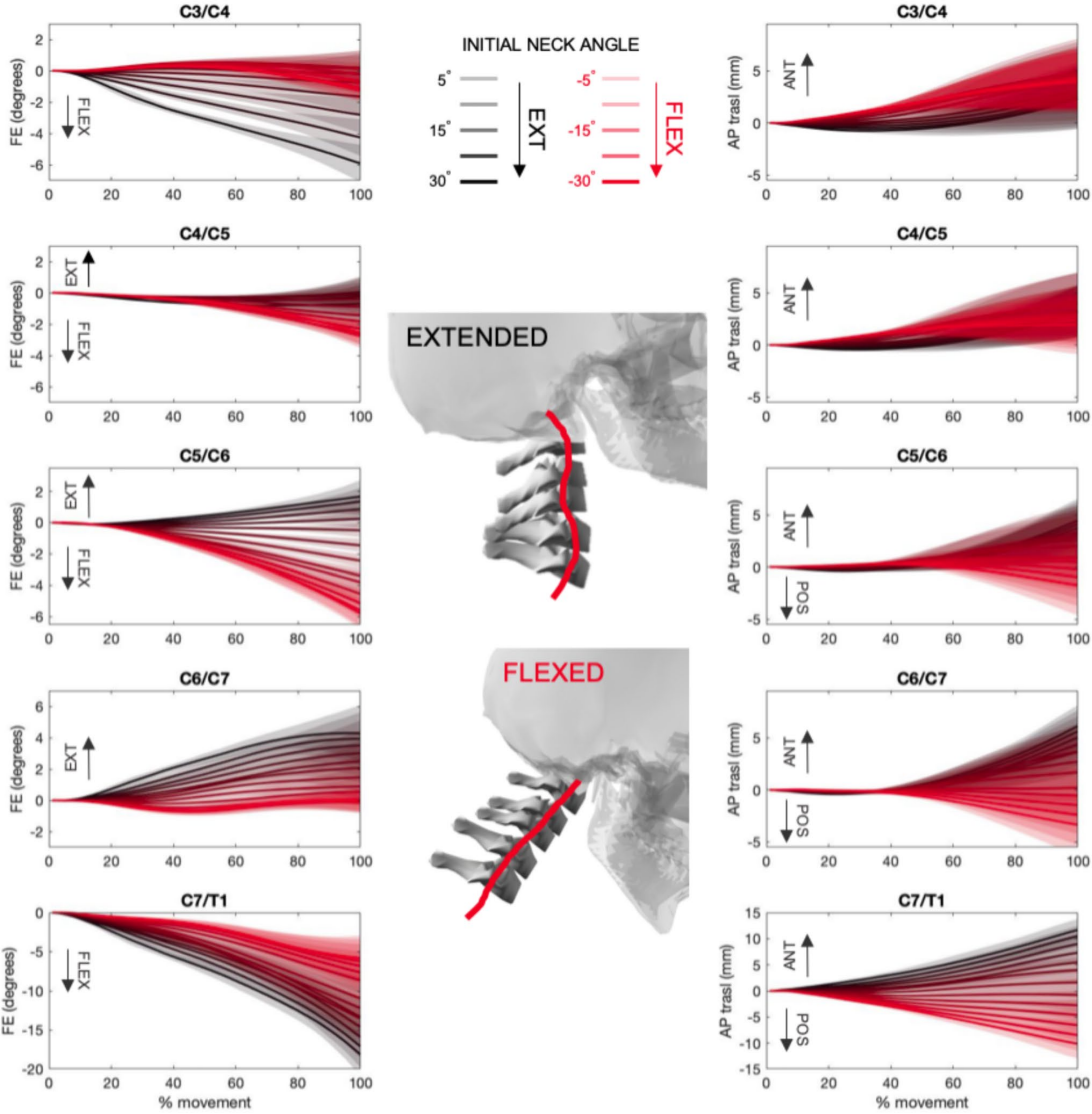
Intervertebral joint rotational (flexion/extension—left) and linear (anteroposterior—right) kinematics curves across all loading conditions. Extension and anterior translation are represented by positive values, while flexion and posterior translation by negative values. Solid lines represent the mean curves and shaded area the standard deviation. The lines and area in black are representative of simulations, where the initial neck angle was in extension. The lines and area in red are representative of simulations, where the initial neck angle was in flexion. A visual representation of the final cervical spine position when the initial neck position was most extended and flexed is showed in the middle of the figure.

In the simulations where the neck was initially flexed, intervertebral sagittal-plane rotational kinematics (flexion/extension) demonstrated that all intervertebral joints below C4 moved into increased flexion (red curves in Fig. 7). In contrast, when the neck was initially extended the neck adopted an ”S-shape,” commonly referred to as the ”second-order buck ling” mechanism [31, 32] (flexion at C3/C4 and C7/T1, extension from C5/C7, and neutral at C4/C5; black curves in Fig. 7). The buckling posture was associated with intervertebral anterior translation at all levels, exceeding physiological limits in the lower cervical spine (C5/T1; Fig. 7. In the simulations where the neck was initially flexed, the lower cervical spine (C6/T1) showed high posterior translations (red curves in Fig. 7), while the mid- and upper cervical spine showed either neutral or slight anterior translation (C3/C4).

## Discussion

In this study, the influence of the initial neck position on the dynamic response of the cervical spine during ”head-first” rugby tackles was investigated using computer simulations. Intervertebral loads and kinematics during impact were influenced by the initial neck flexion-extension angle, but pre-impact lateral bending and axial rotation angles had little effect. An initially flexed neck was forced into additional flexion that was distributed over the entire cervical spine and did not exceed physiological load or motion limits [22]. In contrast, if the neck was in extension at impact, then an “S-shape” or ”buckling” posture was produced, and supraphysiologic intervertebral shear [41] and flexion motions [56] were observed in the lower

cervical spine (Fig. 7). This second-order buckling was characterized by high flexion angles and anterior shear translation in the lower cervical spine (C7/T1), accompanied by high extension angles and anterior shear translation in the mid-cervical spine (C4–C6), and further flexion at the C3/C4 level. These observations are consistent with qualitative descriptions of the spinal pose at injury in previous in vitro [19] and computer simulations [35], supporting the hypothesis that one of the main drivers for catastrophic cervical spine injuries during accidental axial “head-first” rugby impacts is head–neck flexion-extension angle at the instant of impact.

**Fig. 8:**
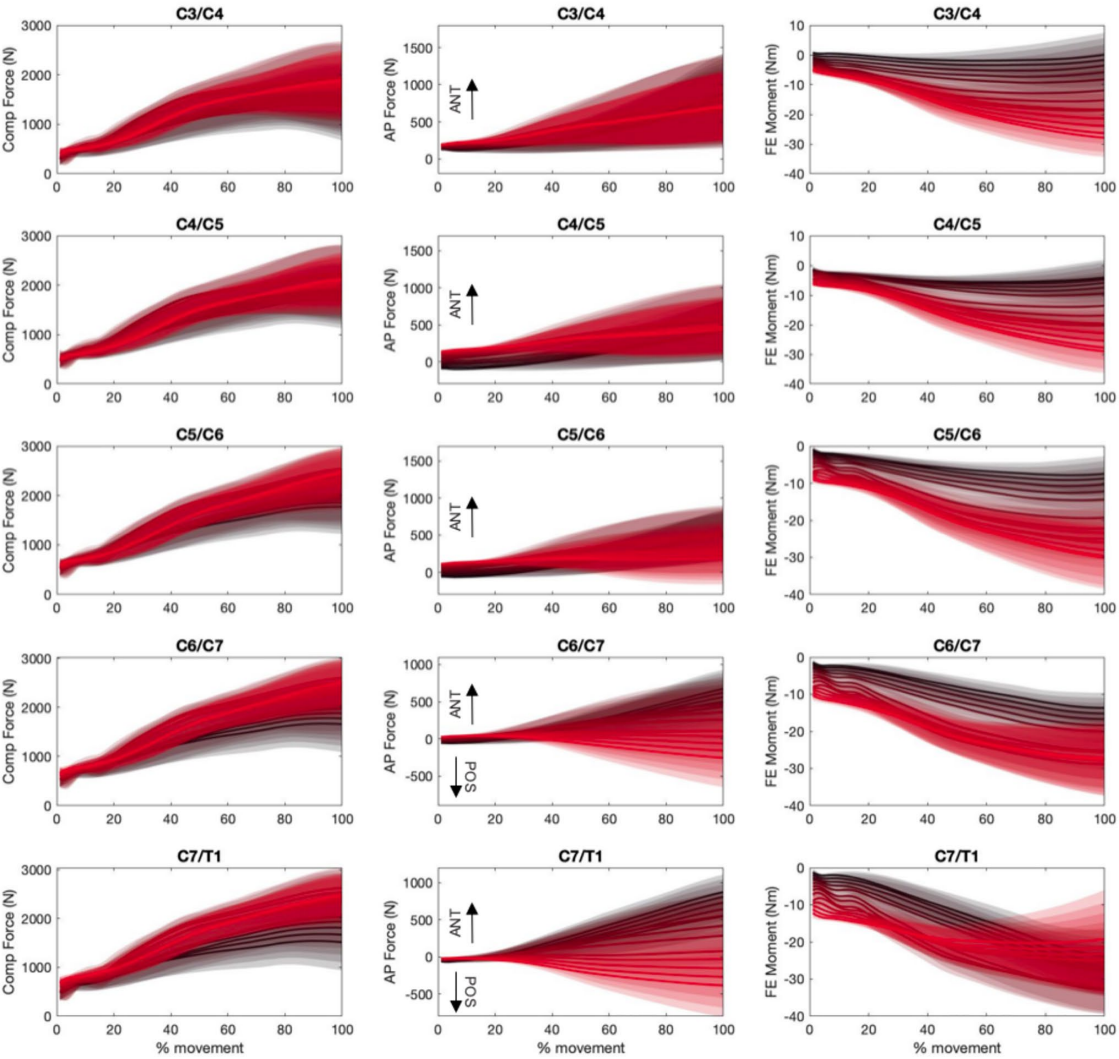
Intervertebral joint kinetics curves including compressive force (left), shear force (center), and flexion/extension moment (right) across all loading conditions. Compressive and anterior forces, and flexion moment are represented by positive values. Solid lines represent the mean curves and shaded area the standard deviation. The lines and area in black are representative of simulations, where the initial neck angle was in extension. The lines and area in red are representative of simulations, where the initial neck angle was in flexion.

Intervertebral compression and flexion loads and kinematics did not exceed injury thresholds when axial impact loads were applied to pre-flexed necks (Fig. 8). A flexed head–neck reduces the natural lordosis of the neck, resulting in an axial alignment of the vertebrae and a stiffer configuration of the cervical spine. This has been reported to increase cervical fracture risk, as axial head impact forces result in compressive loading of both the anterior and posterior columns of the cervical spine [35, 36]. In the current study, compressive joint loads increased, and anterior shear forces decreased, with increasing initial neck flexion as the cervical column aligned with the impact force vector. However, these simulations either resulted in an overall neck angle that exceeded chin-to-chest limits [21], or the peak anterior–posterior [41] and compressive [32] estimated loads were below physiological limits. This result supports the conclusions of in vitro experimental investigations into lower neck anterior dislocation, the most common catastrophic injury in rugby tackling [10, 25], which have mostly shown that an entire cervical spine hyperflexion is not a possible mechanism [27, 31, 32].

In the current study, head impact with an extended neck produced high compression loads, and supraphysiologic anterior shear [41] and flexion [22] loads and motions [32] in the lower cervical spine well before the neck approached the anatomical chin-to-chest limit (40°of flexion) [21]. This supports the hypothesis that catastrophic neck injury, such as facet dislocation, can be produced without approaching or exceeding physiological neck flexion range of motion. Neck buckling produces complex intervertebral motions that can result in injurious loads and motions in the lower cervical spine (C3–C7) [36] without the excessive head motions that may be intuitively indicative of neck injury [31]. This may explain why studies that relate field injuries to video analysis and player recollection of the inciting event support the hyperflexion injury hypothesis, as the instant of injury (which occurs almost immediately following impact [34, 43]) is not externally visible, and is followed by the player’s skull collapsing into the chin-to-chest posture. Computational approaches can complement external injury observations (e.g., from video surveillance studies) and are likely vital for exploring the complexities of neck kinematics and loading associated with catastrophic rugby injuries.

Video analysis has estimated that energy transfer during rugby tackles is between 1.4 and 3.0 kJ [5]. This is considerably greater energy than the 82 J shown to be required to cause neck injury via axial impact in vitro [31] and computer simulations [35]. This highlights the importance of correct tackling technique to position the tackler’s head away from the oncoming ball carrier and minimize the amount of energy transferred to the neck during impact in an accidental head-first tackle. Low tackles (i.e., those targeting the hips and thighs of the ball carrier), which are aimed lateral to the center of mass, are more effective in arresting the ball carrier’s momentum and reduce the possibility of concussion to the tackler, as the tackler’s head is directed away from the ball carrier’s center of mass located within their torso-pelvis. However, this requires the tackler to bend at the waist and, to maintain visibility of the ball carrier, they must subsequently extend their head/neck to raise their gaze. The simulation results indicate that this tackling posture (i.e., head-up) increases the risk of catastrophic neck injuries if accidental head-first contact occurs. Although challenging to model and validate, future computational and experimental studies could evaluate the effects of the tackler’s head contacting more conforming body regions such as the abdomen, groin, or thigh, compared to more rigid regions such as those overlying the ribs or pelvis. Head contact in areas with less overlying soft tissue may result in more glancing impacts with high head acceleration and risk of concussion, compared with conforming areas with soft tissue and organs [34]. In the future, injury prevention studies should focus on including such contact situations and brain computational models to provide a complete overview on the effect of tackle height to both concussive head and catastrophic neck injuries. The important role of active and passive neck muscle forces to load the cervical spine during impacts has been investigated [8, 11, 16, 35]. Neck musculature provides a compressive preload that increases the stability of the spinal column but also brings the intervertebral loads closer to the passive structure and vertebral critical failure limits [43]. In this study, muscle-driven [46] simulations of head-on impacts permitted the use of physiologically plausible neck muscle forces at the time of impact. The simulation results replicated the injury patterns produced in in vitro studies, further demonstrating the biofidelity of the musculoskeletal model [47] and neural recruitment strategy [46] used.

There are limitations related to the methodological approach used. Firstly, the factors associated with the injury severity of a head-first rugby tackle are many [34, 43], and all possible factor combinations that might be experienced on a rugby field cannot be fully replicated experimentally or computationally. Player internal factors such as experience, physical maturity, fitness, and technique [40, 52] and inciting event characteristics, such as the tackler and ball carrier approach velocities, will all influence the risk of vertebral injury. This study was conducted using an MRI-informed participant-specific musculoskeletal model of a rugby player with prescribed task-specific body kinematics and muscle forces. A limitation of this approach is that neck muscle forces and initial angular velocities applied across all conditions were estimated from a single experimental neck position during a tackle. Ideally, for each initial neck angle condition simulated in this study, muscle activations would have been experimentally estimated or optimized based on an a priori criterion. However, this was experimentally infeasible and outside the scope of this study.

The loading conditions selected aimed to replicate impact directions and locations representative of head-first tackles. In reality, impacts to the head would result in a shear force component, and a consequent translation of the point of force application that would change the vector of the applied force over the duration of the event. This variation in applied load is difficult to replicate in multibody models. For this reason, it was assumed that a point load would be a reasonable representation for the short duration simulated (50 ms), as has been done in the previous musculoskeletal studies [24, 29]. In the future, validated contact models should be developed and validated to examine the effect of tackling technique (e.g., neck and relative torso angles, loading condition) on neck loading, also including the thoracic spine in the multibody models to provide more appropriate boundary conditions to the caudal end.

The response of the cervical spine to axial head impacts has been explored previously with simplified finite element models that included rigid vertebrae and beam elements to represent the overall intervertebral joint behavior [35], similar to the bushing elements used in the present study. This approach may cause artifacts in the anterior–posterior direction, such as the high posterior translations (red curves in Fig. 7) of the lower cervical spine, and highlights the need to integrate facet joint contact models.

Although musculoskeletal models cannot simulate injury, under controlled circumstances, they provide a more streamlined solution to perform data-driven simulations and investigate the cervical spine’s response to external impacts. The simulations herein did not continue past the peak external loading at 50 ms because past this point plastic deformation (or injury) is likely to occur [33]. In fact, to fully understand the cause–effect relationship between the internal loading, the resultant kinematic response of the cervical spine, and clinically observed injuries during accidental head-first rugby tackles, a further step using finite element models should be completed. In vivo kinematics (vertebral alignment, joint angular velocities, and segmental accelerations) and muscle forces predicted by the musculoskeletal model could be used as boundary conditions in detailed finite element simulations to then identify localized regions of stress and strain on cervical spine structures. These finite element models could provide the specific identifiers of how cervical spine buckling leads to injury on a local vertebral level (e.g., facet joint dislocation, vertebral fracture, ligament, or disc rupture).

## Conclusion

This study evaluated the effect of pre-impact head–neck posture on cervical spine kinetics and kinematics during head-first misdirected rugby tackles. In the simulations, an initially flexed neck increased axial compression forces and increased flexion angles without exceeding injury thresholds. In contrast, an extended neck caused supraphysiologic intervertebral shear and flexion loads and motions in the lower cervical spine. This loading pattern is observed in the most common rugby-related catastrophic neck injury, bilateral facet dislocation. These findings highlight the importance of adopting a correct tackling technique and inclusion of biomechanical analyses to inform injury prevention strategies and ensure the safety of the athletes in rugby.
